# Supplementary material for: Expanded palette of RNA base editors for comprehensive RBP-RNA interactome studies
Source: Nat Commun. 2024 Jan 29;15:875. doi: 10.1038/s41467-024-45009-4 (PMC10825223; doi:10.1038/s41467-024-45009-4)
Supplement: Supplementary file 1 — Supplementary Information [file 41467_2024_45009_MOESM1_ESM.pdf]

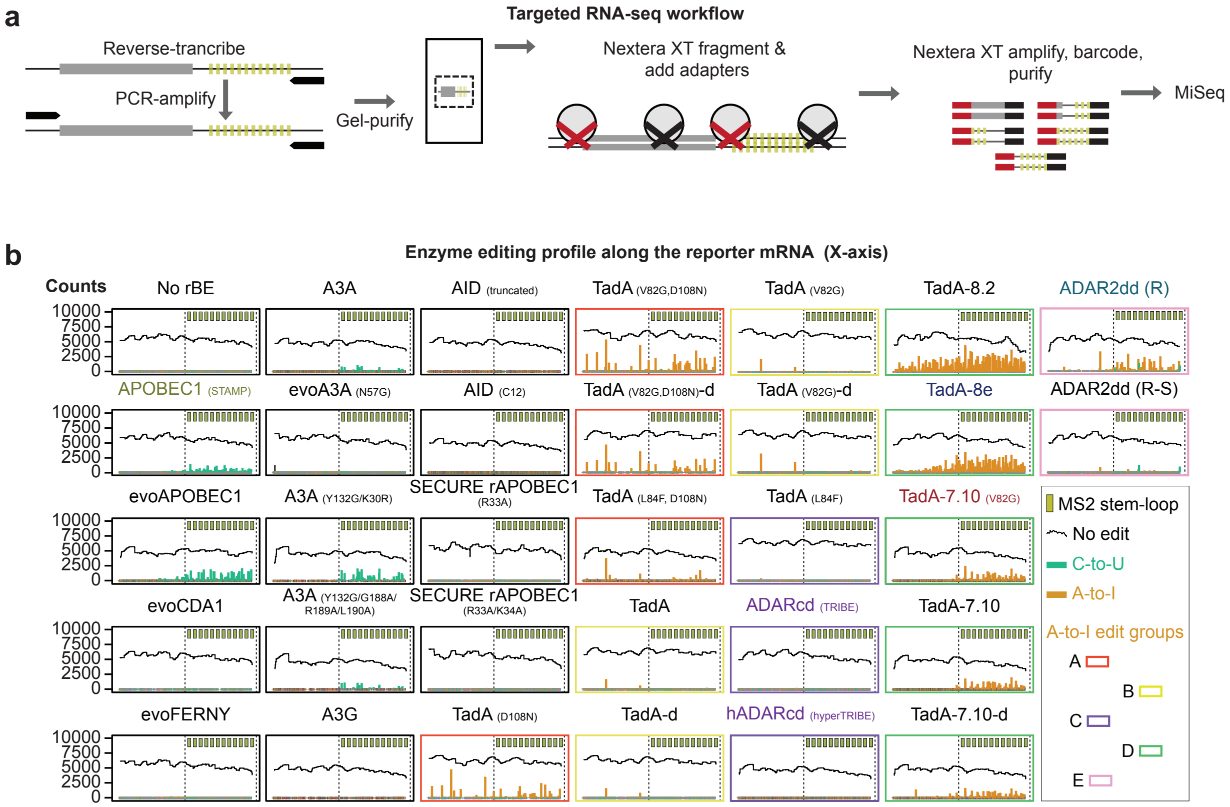

Supplementary Figure 1: Workflow and enzyme-specific RNA editing profiles in targeted RNA-sequencing experiments.

a) Targeted RNA-seq strategy to detect edits along the reporter mRNA sequence.

b) The number of times a base was called (y-axis) at each position along the twelve-MS2 stem-loop reporter construct (as in Figure 1). The fraction of each position in the reporter that contained either a cytosine (C) but a uridine was detected (C-to-U) or an adenosine (A) but an inosine was detected (read as guanosine, A-to-I) are denoted by green and orange bars, respectively. Positions where the called base matches the reporter sequence (no edit) are indicated by a black line.

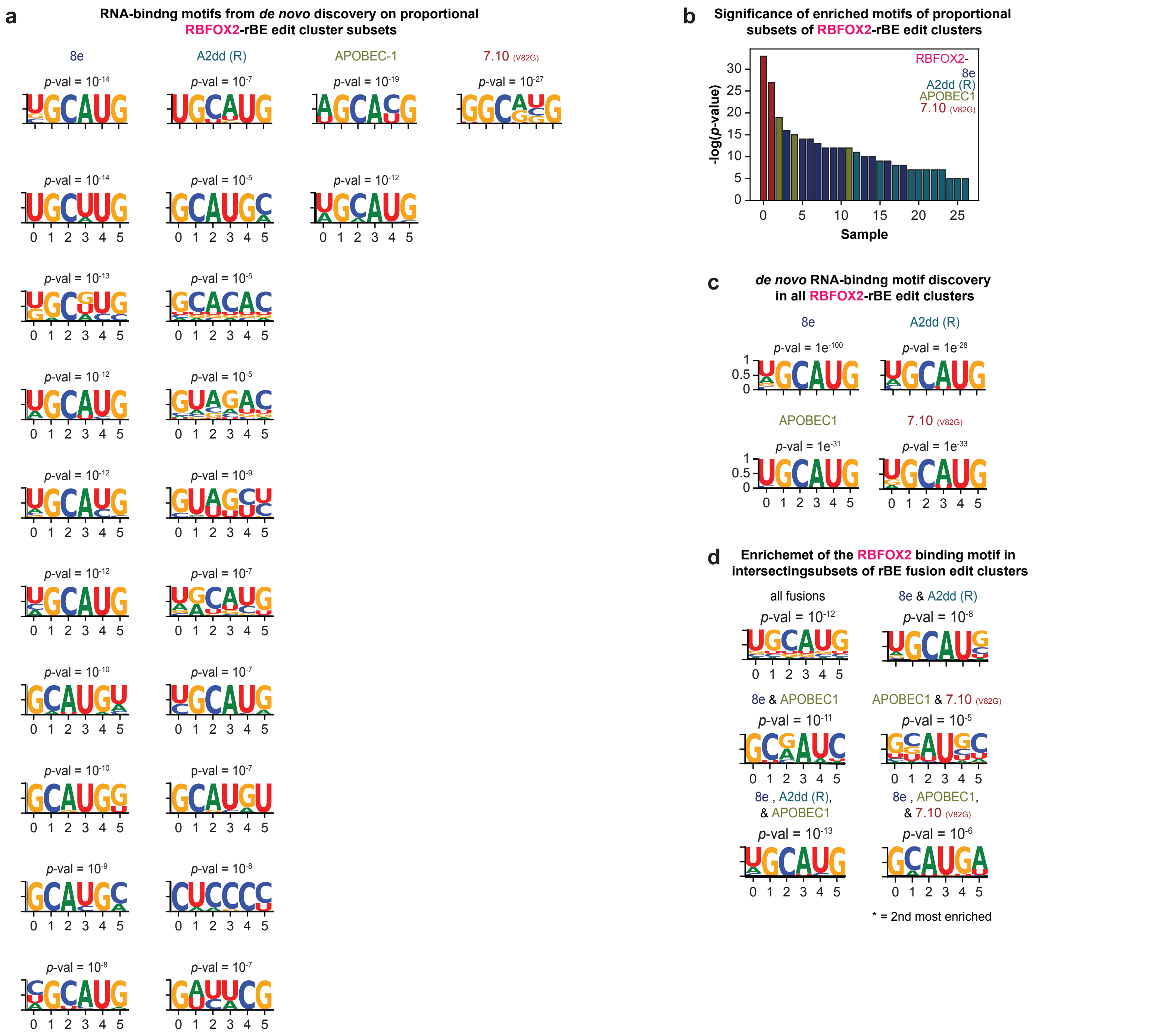

Supplementary Figure 2: Identification and analysis of RNA-binding protein motifs and enrichment in RNA editing experiments.

a) HOMER motif enriched in an equal number of edit clusters sampled from each RBFOX2-rBE fusion set. 736 edit clusters were repeatedly selected from each cluster set at random and without replacement until no further sets of 736 clusters could be chosen. The most enriched sequence motif produced by a selection of 736 clusters from each fusion is shown in Figure 2e, and the motifs for selections that yielded lower p-values are shown here in order of most (top) to least (bottom) significant. HOMER assigns p-values to motif enrichment compared to GC-matched background sequences using a cumulative hypergeometric distribution.

b) The negative logarithm-transformed p-values (-log p-value) associated with the HOMER motifs listed in Supplementary Figure 2a.

c) HOMER detects the RBFOX2 motif among each set of RBFOX2-rBE edit clusters.

d) The RBFOX2 binding motifs are the most or second most (*) enriched HOMER motif among each set of intersecting peaks (see also Figure 2i).

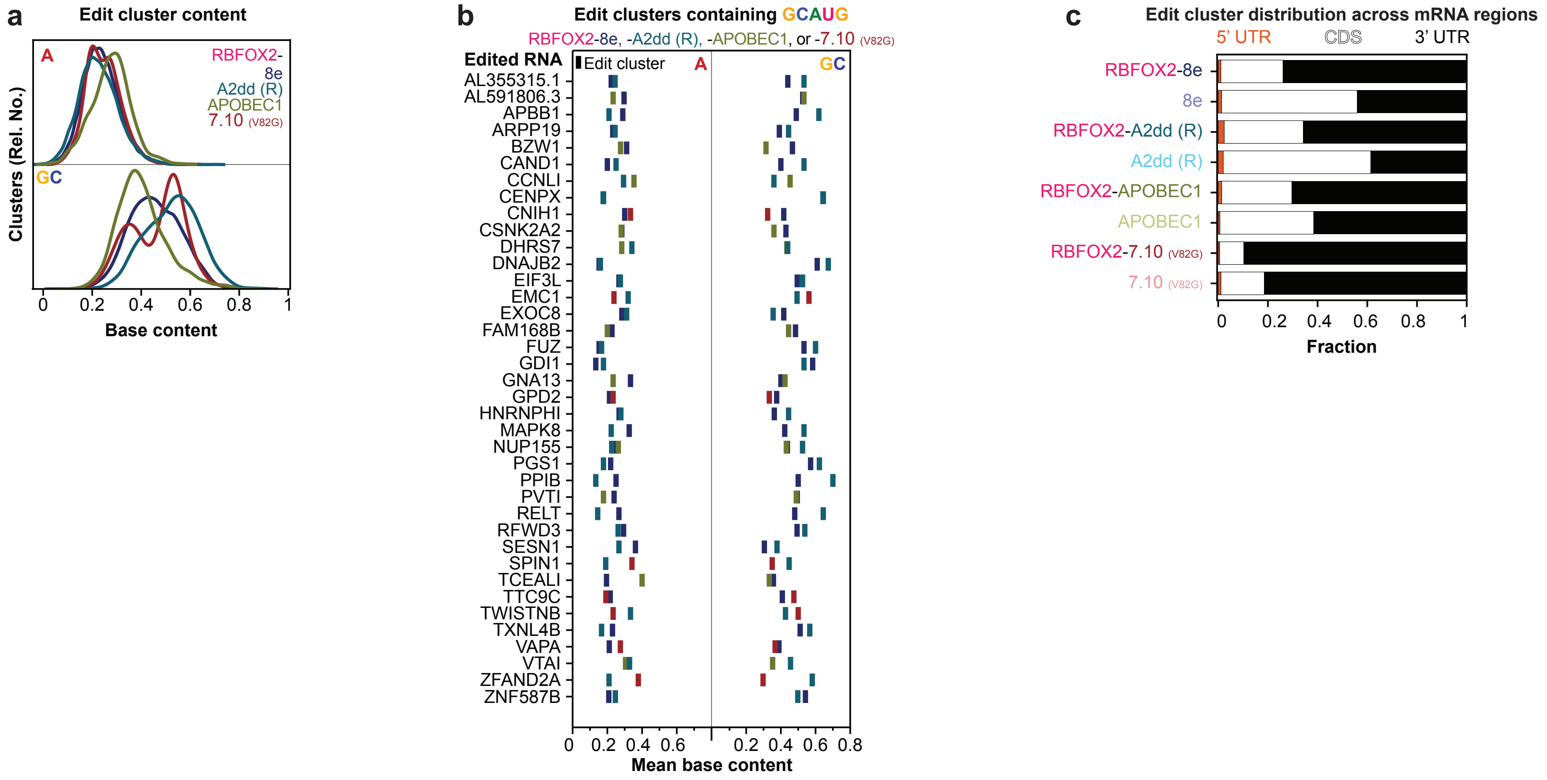

Supplementary Figure 3: Content and distribution analysis of RNA editing sites in relation to RBFOX2 binding motifs and mRNA regions.

a) A density plot of the overall peak adenosine (A, top) and guanosine-cytidine (GC, bottom) content for RBFOX2-rBE fusions before subtraction of the edit clusters produced by the corresponding free rBE construct (see Figure 3e for density plots of the remaining RBFOX2-rBE and all free rBE edit clusters). The various fusions are distinguished using unique colors. The fusion of RBFOX2 with 8e is depicted in dark blue. When RBFOX2 is fused with A2dd (R), it's represented in teal. The fusion between RBFOX2 and APOBEC1 appears in green, and the RBFOX2 fusion with 7.10 (V82G) is illustrated in red.

b) On genes with two GCAUG core motif-containing clusters edited by two different enzymes, the combination of flanking window base contents and enzyme editing context specificities depicted in panel E dictate which region is more likely to be edited by each RBFOX2-rBE fusion.

c) Analyses of where on the mRNA region the RBFOX2-rBE and free rBE edit clusters map. Bars demonstrate the fraction of edit clusters that map to the 5' (red) or 3' (black) untranslated region (UTR), and those mapping to the coding sequence (CDS, white). The labels follow the color scheme outlined in Supplementary Figure 3a for each RBFOX2-rBE fusion. For enzymes that are not fused with RBFOX2, we've assigned unique colors: 8e is in purple, A2dd (R) in turquoise, APOBEC1 in light green, and 7.10 (V82G) in pink.

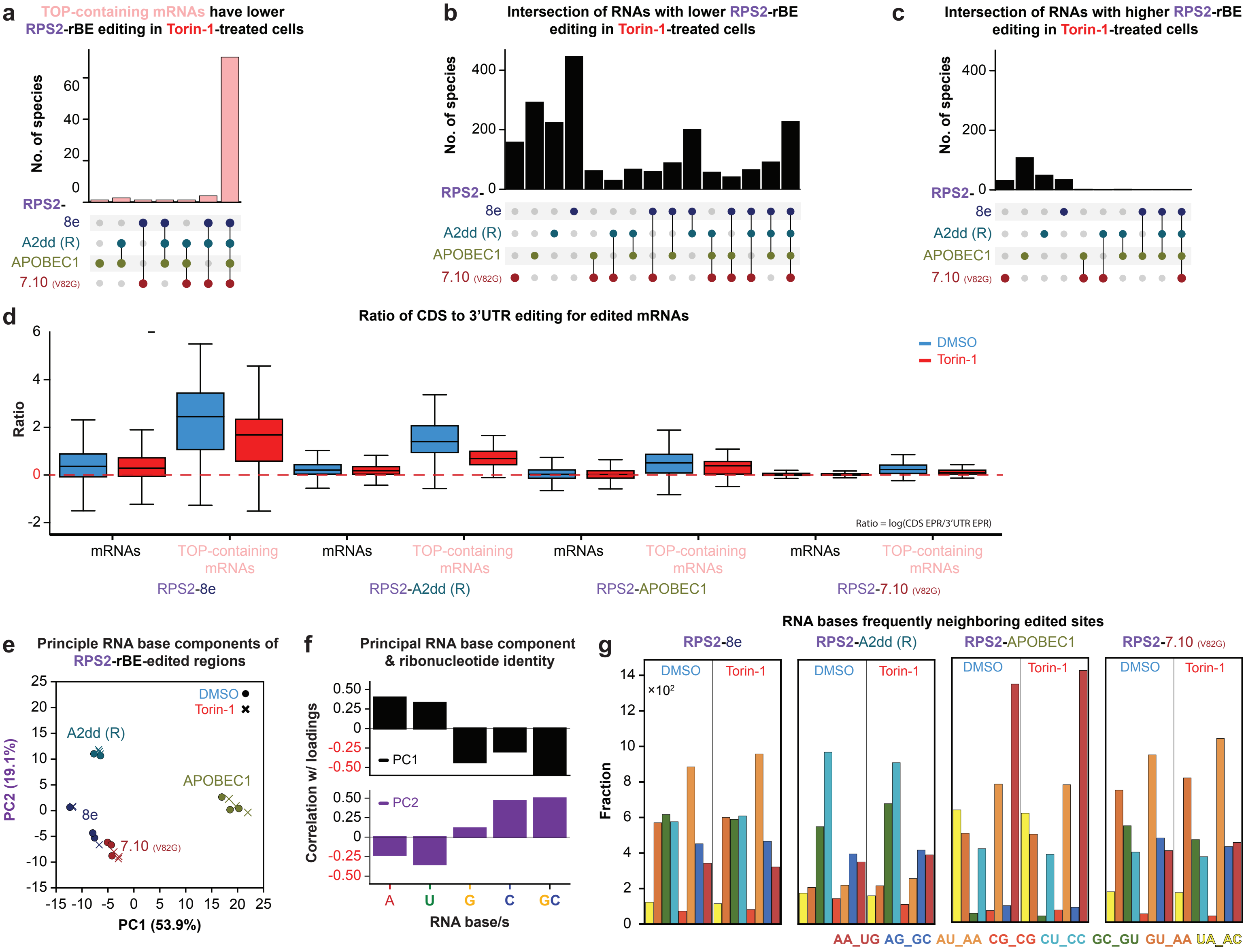

Supplementary Figure 4: Impact of Torin-1 on RPS2-rBE RNA editing patterns and distribution (see Methods).
a) - c) The intersection between RBFOX2-rBE constructs for a) TOP-containing (pink bars) and b) all edited poly(A)+ RNAs (black) that experienced a lower EPR in Torin-1-treated cells relative to the DMSO vehcle-treated cells, and c) all poly(A)+ RNAs (black) with an increased EPR. The dots are colored uniquely to represent rBE fusions to RBFOX2. Fusion with 8e is depicted in dark blue. When RBFOX2 is fused with A2dd (R), it's represented in teal. The fusion between RBFOX2 and APOBEC1 appears in green, and the RBFOX2 fusion with 7.10 (V82G) is illustrated in red. Vertical lines bisect colored dots to connect RBFOX2-rBEs with intersecting values, and single-colored dots without a line indicate unique clusters for the respective RBFOX2-rBE.
d) The ratio of RPS2-rBE edits between mRNA coding sequences (CDSs) and 3' untranslated regions (3'UTRs) for TOP-containing (pink letters) and all poly(A)+ RNAs (black letters). The plot boxes are colored based on whether the cells were grown in uninhibited (DMSO, blue) or translation-inhibiting (Torin-1-treated, red) conditions. Boxplot boxes extend from the first to the third quartile of the data, with the center line indicating the median. Box whiskers extend to the farthest data point lying within 1.5x the inter-quartile range from the box in either direction.
e) PCA analysis of bases flanking edited sites for RBFOX2-rBE fusions. The X denotes the Torin-1-treated samples, while the circles denote samples treated with DMSO vehicle. These symbols are colored in a manner described in Supplementary Figure 3c for RBFOX2-rBE fusions.
f) Contribution of each RNA base (A, U, G, or C) or combination (GC) to principal components PC1 (black) and PC2 (purple).
g) The top pairs of RNA bases flanking sites edited by each RPS2-rBE fusion. The bars denote the fraction of the edited sites within a given context, and the bars are colored in agreement with the corresponding sequence context listed at the bottom.

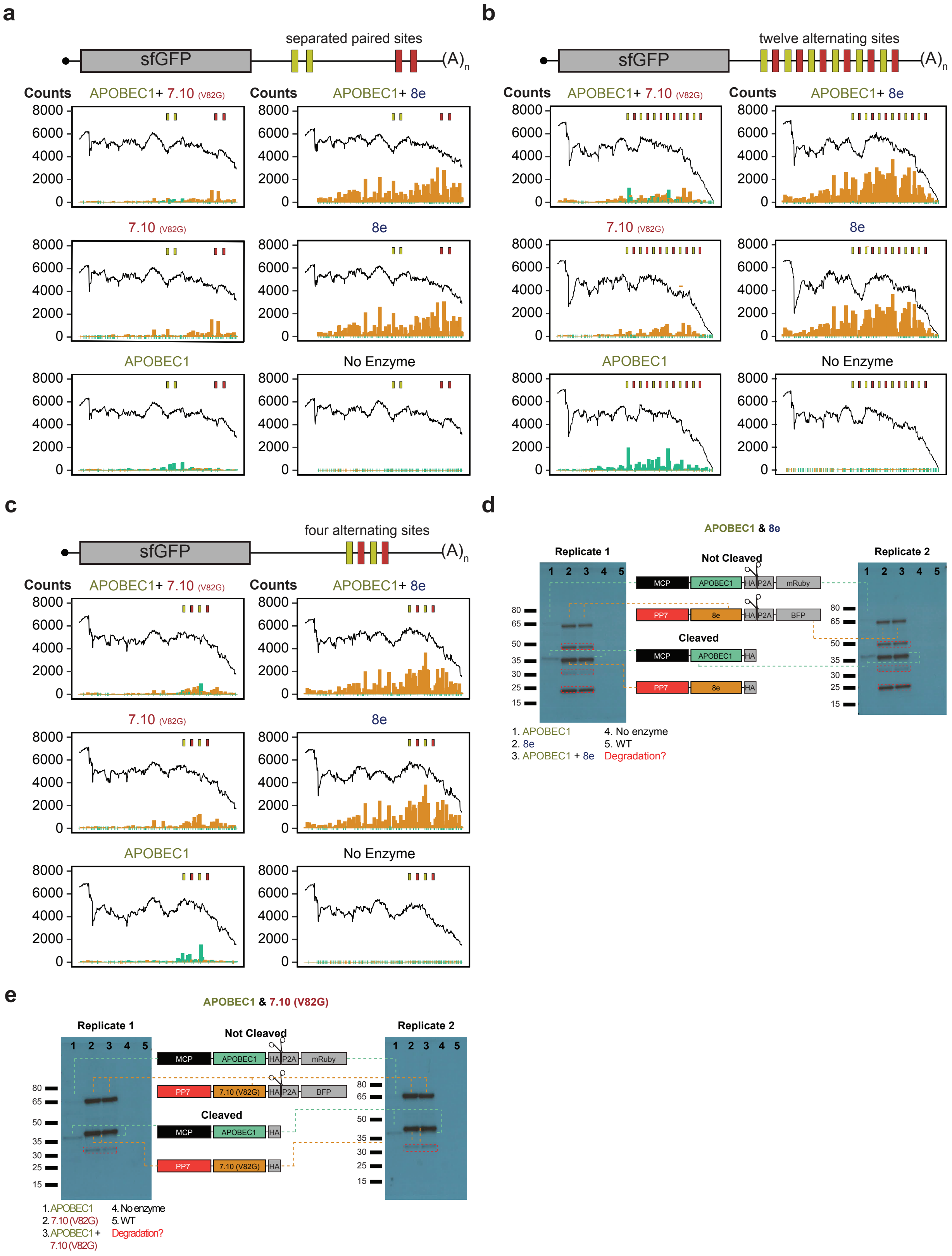

Supplementary Figure 5: Replicate combinatorial editing profiles and protein expression analysis.
As in Figure 5, the combinatorial editing profiles for MCP-APOBEC1 together with either PP7-CP-8e or PP7-CP-7.10 (V82G) when they are co-expressed in HEKs with a reporter mRNA bearing MS2 (yellow bar) and PP7 (red bar) stem-loops in **a)** split pairs or **b)** twelve and **c)** four alternating sites. The fraction of total covered bases at each position along each reporter exhibiting either C-to-U (green) or A-to-I (orange) are denoted as bars. In contrast, the fraction of reads with no edits is denoted by a black line. **d) & e)** Western blot analysis was conducted for MCP-APOBEC1 in combination with either PP7-CP-8e or PP7-CP-7.10 (V82G), as well as for each expressed individually. This analysis was replicated in n = 2 independent experiments. Corresponding to each gel, the lanes are numbered 1 to 5, with each experiment's number indicated next to its respective lane. Beside the gels, diagrams of the constructs are provided, illustrating the expected product represented by each band in the blot.
