# Supplementary material for: Expanded palette of RNA base editors for comprehensive RBP-RNA interactome studies
Source: Nat Commun. 2024 Jan 29;15:875. doi: 10.1038/s41467-024-45009-4 (PMC10825223; doi:10.1038/s41467-024-45009-4)
Supplement: Supplementary file 3 — Reporting Summary [file 41467_2024_45009_MOESM3_ESM.pdf]

nature portfolio

Corresponding author(s): Yeo, Gene

Last updated by author(s): 2023/11/23

Reporting Summary

Nature Portfolio wishes to improve the reproducibility of the work that we publish. This form provides structure for consistency and transparency in reporting. For further information on Nature Portfolio policies, see our Editorial Policies and the Editorial Policy Checklist.

Statistics

For all statistical analyses, confirm that the following items are present in the figure legend, table legend, main text, or Methods section.

n/a	Confirmed	
☐	☒	The exact sample size (*n*) for each experimental group/condition, given as a discrete number and unit of measurement
☒	☐	A statement on whether measurements were taken from distinct samples or whether the same sample was measured repeatedly
☐	☒	The statistical test(s) used AND whether they are one- or two-sided<br>*Only common tests should be described solely by name; describe more complex techniques in the Methods section.*
☒	☐	A description of all covariates tested
☐	☒	A description of any assumptions or corrections, such as tests of normality and adjustment for multiple comparisons
☐	☒	A full description of the statistical parameters including central tendency (e.g. means) or other basic estimates (e.g. regression coefficient) AND variation (e.g. standard deviation) or associated estimates of uncertainty (e.g. confidence intervals)
☐	☒	For null hypothesis testing, the test statistic (e.g. *F*, *t*, *r*) with confidence intervals, effect sizes, degrees of freedom and *P* value noted<br>*Give P values as exact values whenever suitable.*
☒	☐	For Bayesian analysis, information on the choice of priors and Markov chain Monte Carlo settings
☒	☐	For hierarchical and complex designs, identification of the appropriate level for tests and full reporting of outcomes
☒	☐	Estimates of effect sizes (e.g. Cohen's *d*, Pearson's *r*), indicating how they were calculated

Our web collection on statistics for biologists contains articles on many of the points above.

Software and code

Policy information about availability of computer code

Data collection	The sequencing data were collected using the MiSeq and NovaSeq 6000 platforms, operated with MiSeq Software version 4.0 and NovaSeq Software version 1.7.5, respectively.
Data analysis	The software utilized and code generated for the analyses in this study can be obtained by accessing the following repositories: https://snakemake.readthedocs.io/en/v5.6.0/getting_started/installation.html, and https://github.com/YeoLab/PRINTER

For manuscripts utilizing custom algorithms or software that are central to the research but not yet described in published literature, software must be made available to editors and reviewers. We strongly encourage code deposition in a community repository (e.g. GitHub). See the Nature Portfolio guidelines for submitting code & software for further information.

Data

Policy information about availability of data

All manuscripts must include a data availability statement. This statement should provide the following information, where applicable:

- Accession codes, unique identifiers, or web links for publicly available datasets
- A description of any restrictions on data availability
- For clinical datasets or third party data, please ensure that the statement adheres to our policy

Raw and assembled sequencing data from this study is available in NCBI's Gene Expression Omnibus (GEO) under accession code GSE232520. The RBFOX2-APOBEC1

data used in this study are available in the GEO database under accession code GSE215252. The sequencing data generated in this study were collected using MiSeq Software v.4.0 or NovaSeq Software v.1.7.5.

Research involving human participants, their data, or biological material

Policy information about studies with human participants or human data. See also policy information about sex, gender (identity/presentation), and sexual orientation and race, ethnicity and racism.

Reporting on sex and gender	NA
Reporting on race, ethnicity, or other socially relevant groupings	NA
Population characteristics	NA
Recruitment	NA
Ethics oversight	NA

Note that full information on the approval of the study protocol must also be provided in the manuscript.

Field-specific reporting

Please select the one below that is the best fit for your research. If you are not sure, read the appropriate sections before making your selection.

☒ Life sciences ☐ Behavioural & social sciences ☐ Ecological, evolutionary & environmental sciences

For a reference copy of the document with all sections, see nature.com/documents/nr-reporting-summary-flat.pdf

Life sciences study design

All studies must disclose on these points even when the disclosure is negative.

Sample size	The screens conducted with the 3’ 12X MS2 stem-loop reporter mRNA and MCP-rBE fusions were performed in duplicate. This decision was based on the high reproducibility observed in initial tests with APOBEC1, and, as anticipated, the results were highly reproducible across all rBEs tested.<br><br>For the RBP fusion experiments, we opted for triplicate runs. Although previous studies, such as Brennan et al. (2021), reported that two replicates were adequate for detecting RBP-mediated editing, we found that using three replicates enhanced the correlation with RBFOX2 eCLIP datasets. This observation aligns with our previous findings, as detailed in Kofman et al. (2023), demonstrating the added value of a third replicate in capturing more comprehensive data.
Data exclusions	Paired-end sequencing was employed for some samples, although only Read 1 was analyzed due to the prevalence of single-end sequencing in most of the experiments. Paired-end sequencing was chosen in cases where it was more economical or efficient. Both reads, when applicable, will be submitted to the Gene Expression Omnibus (GEO) repository.
Replication	In our reporter-based assays, we conducted two replicates per experiment, a decision supported by strong reproducibility observed in our preliminary studies. For the RBP fusion experiments, we increased this to three replicates per experiment, focusing our analysis specifically on genes that were consistently detected across all replicates. This approach, as we anticipated, demonstrated the high level of reproducibility we expected, both in specific assays and across the broader experimental framework.
Randomization	N/A. All experiments were conducted using the same cell line.
Blinding	N/A. All experiments were conducted using the same cell line.

Reporting for specific materials, systems and methods

We require information from authors about some types of materials, experimental systems and methods used in many studies. Here, indicate whether each material, system or method listed is relevant to your study. If you are not sure if a list item applies to your research, read the appropriate section before selecting a response.

Materials & experimental systems

n/a	Involved in the study
☐	☒ Antibodies
☐	☒ Eukaryotic cell lines
☒	☐ Palaeontology and archaeology
☒	☐ Animals and other organisms
☒	☐ Clinical data
☒	☐ Dual use research of concern
☒	☐ Plants

Methods

n/a	Involved in the study
☒	☐ ChIP-seq
☒	☐ Flow cytometry
☒	☐ MRI-based neuroimaging

Antibodies

Antibodies used	Rabbit anti-HA-tag (1:1,000, Cell Signaling, Cat # 3724, Clone C29F4, Lot 10) and Rabbit TrueBlot: Anti-Rabbit IgG HRP (1:4,000, Rockland Immunochemicals, Cat # 18-8816-33, Clone eB182, Lot 46967).
Validation	Cell Signaling validates their antibodies through Western Blot analysis of selected proteins modified with the HA epitope, while Rockland Immunochemicals validates their antibodies by probing Rabbit IgG-bound proteins. Both companies confirm the sensitivity of detection in a range of cell types.

Eukaryotic cell lines

Policy information about cell lines and Sex and Gender in Research

Cell line source(s)	The Lenti-X Human Embryonic Kidney (HEK293XT) cell line utilized in this study was derived from a female human embryo.
Authentication	Lenti-X 293T Cell Line (Takara Inc.; Cat 632180) authenticated using short tandem repeat (STR) analysis. We authenticate the cell line through morphological analysis using microscopy.
Mycoplasma contamination	Although the cell lines in our tissue culture facility are subject to routine Mycoplasma testing, the specific cultures utilized in these experiments were not subjected to such testing.
Commonly misidentified lines (See ICLAC register)	No commonly misidentified cell lines were used in this study.

Plants

Seed stocks	NA
Novel plant genotypes	NA
Authentication	NA
