# Supplementary material for: Expanded palette of RNA base editors for comprehensive RBP-RNA interactome studies
Source: Nat Commun. 2024 Jan 29;15:875. doi: 10.1038/s41467-024-45009-4 (PMC10825223; doi:10.1038/s41467-024-45009-4)
Supplement: Supplementary file 2 — Peer review file [file 41467_2024_45009_MOESM2_ESM.pdf]

nature portfolio

Peer Review File

Expanded palette of RNA base editors for comprehensive RBP-RNA interactome studies

Open Access This file is licensed under a Creative Commons Attribution 4.0 International License, which permits use, sharing, adaptation, distribution and reproduction in any medium or format, as long as you give appropriate credit to the original author(s) and the source, provide a link to the Creative Commons license, and indicate if changes were made. In the cases where the authors are anonymous, such as is the case for the reports of anonymous peer reviewers, author attribution should be to 'Anonymous Referee' followed by a clear attribution to the source work. The images or other third party material in this file are included in the article's Creative Commons license, unless indicated otherwise in a credit line to the material. If material is not included in the article's Creative Commons license and your intended use is not permitted by statutory regulation or exceeds the permitted use, you will need to obtain permission directly from the copyright holder. To view a copy of this license, visit http://creativecommons.org/licenses/by/4.0/.

REVIEWERS' COMMENTS

Reviewer #2 (Remarks to the Author):

The authors have done a thorough job addressing my concerns. I am still not satisfied with the fact that the authors only tested the combination of C-to-U and A-to-I rBEs in reporter constructs and not in real cells, but I am okay with not including this in this manuscript.

Reviewer #3 (Remarks to the Author):

The authors have addressed and satisfied all raised comments and concerns. I recomment publication of this manuscript that will be a useful resource for the RNA field.
